# The daily life of patients with dementia: A comparative study between the information provided by the caregiver and direct patient assessment

**DOI:** 10.1590/S1980-57642008DN10300011

**Published:** 2007

**Authors:** Lucia Aparecida Bressan, Francisco de Assis Carvalho Vale, José Geraldo Speciali

**Affiliations:** 1 Terapeuta Ocupacional Doutora em Neurociências - Programa de Pós-Graduação em Neurologia. Departamento de Neurologia, Psiquiatria e Psicologia Médica, Faculdade de Medicina de Ribeirão Preto, Universidade de São Paulo (FMRP-USP) Ribeirão Preto SP, Brasil; 2 Doutor em Neurologia, Coordenador do Grupo de Neurologia Comportamental do HCFMRP-USP. Departamento de Neurologia, Psiquiatria e Psicologia Médica, Faculdade de Medicina de Ribeirão Preto, Universidade de São Paulo (FMRP-USP) Ribeirão Preto SP, Brasil.; 3 Professor Associado do Departamento de Neurologia, Psiquiatria e Psicologia Médica. Departamento de Neurologia, Psiquiatria e Psicologia Médica, Faculdade de Medicina de Ribeirão Preto, Universidade de São Paulo (FMRP-USP) Ribeirão Preto SP, Brasil.

**Keywords:** dementia, functional assessment and occupational therapy

## Abstract

The functionality concept is very important, as the diagnosis of dementia presupposes the existence of functional impairment. Instruments assessing functional performance present some limitations. In most cases, the assessment is based on the caregiver’s report. Some studies in international literature have evaluated this issue and concluded that a difference exists between the caregiver’s report and direct patient assessment. American and European caregivers tend to underestimate the patient’s functional limitations. However, this issue has hitherto not been investigated in our context.

**Objective:**

To compare the caregiver’s information with direct assessment of the patient’s performance based on the same functional assessment questionnaire.

**Methods:**

Seventy-two patients and caregivers were attended by the Occupational Therapy service of the Behavioral Neurology Outpatient Clinic between 1999 and 2001, 25 of whom fulfilled the inclusion criteria: having a confirmed diagnosis of dementia according to the DSM-IV; having attended three or more return appointments, and where the caregiver belonged to the patient’s family nucleus. The remaining subjects were excluded because of non-adherence to treatment or refusal to participate in the study. The Functional Activities Questionnaire by Pfeffer et al., 1982 was applied to patients in a laboratory simulation, while another evaluator interviewed the respective caregivers. The data were analyzed based on the weighted Kappa coefficient, and Wilcoxon test.

**Results:**

There were significative differences between caregiver’s answers and direct observation of the patient’s performance. The information provided by the caregivers proved unreliable since caregivers underestimated the patient’s functional capacity.

## Abstract (pt)

O conceito de funcionalidade se reveste de grande importância, uma vez que, o diagnóstico de Demência pressupõe a existência de comprometimento funcional. Os instrumentos que avaliam o desempenho funcional apresentam algumas limitações, na maioria das vezes, é feita a partir do relato do cuidador. Alguns estudos da literatura internacional avaliaram esta questão e concluíram que existe diferença entre o relato do cuidador e a avaliação direta do paciente. O cuidador americano e europeu tende a minimizar as limitações funcionais do paciente. No entanto, essa questão não foi investigada em nosso meio até o momento.

**Objetivo:**

Comparar as informações do cuidador com avaliação direta do desempenho do paciente, frente a um mesmo questionário de avaliação funcional.

**Métodos:**

Dos 72 pacientes e cuidadores atendidos pela Terapia Ocupacional do Ambulatório de Neurologia Comportamental no período entre 1999 e 2001, um número de 25 pacientes e cuidadores atenderam aos critérios de inclusão: ter diagnóstico confirmado de demência segundo o DSM-IV; ter comparecido a três retornos ou mais e o cuidador pertencer ao seu núcleo familiar. Os demais foram excluídos pela não-aderência ao tratamento ou recusa em participar do estudo. O Questionário de Atividades Funcionais Pfeffer et al., 1982 foi aplicado, em situação simulada em laboratório, enquanto outro avaliador entrevistava os respectivos cuidadores. Os dados foram analisados com base no coeficiente Kappa ponderado e teste de Wilcoxon.

**Resultados:**

Foram encontradas diferenças significativas entre as respostas do cuidador e a observação direta do desempenho do paciente. As informações fornecidas pelos cuidadores não são fidedignas. O cuidador subestima a capacidade funcional do paciente.

The main risk factor for many chronic diseases occurring with aging is indeed age itself. Disease-free aging is the privilege of few individuals, but aging with health can be achieved by many.

The most prevalent chronic-degenerative disease in the elderly is dementia.

The concept of functionality is very important in the treatment of dementia since the disease diagnosis is based on the assumption of the existence of functional impairment. Functionality is understood as the ability of an individual to maintain the physical and mental skills necessary to lead an independent and autonomous life.^1^ Functional capacity, considered to be a new health paradigm, requires an expansion of the prevention, assistance and rehabilitation actions aimed at the health of the elderly in order to improve or maintain functional performance.

The instruments that evaluate functionality have some limitations. For example, disabilities may or may not be observed depending on the activities performed by the elderly. Another very important limitation is related to the fact that, in most cases, the evaluation is exclusively based on the information given by the person accompanying the patient, which may introduce bias into the assessment.

Some studies have addressed this issue and the key conclusions reached were that caregivers overestimate the ability of the patient to perform daily activities^2^ and that depression interferes with the evaluation of the caregiver, whereas care load does not.^2^ In contrast, other studies have stated that depressive symptoms do not interfere with the difference between evaluations^3^ whilst care load does.^4^

In Brazil, the use of the Pfeffer Functional Activity Questionnaire (FAQ)^5^ has been recommended for the diagnosis of dementia.^6^ Scales such as the FAQ (1982), the scale applied to the informant, the Informant Questionnaire of Cognitive Decline in the Elderly” (IQCODE), and the Bayer Scale of Activities of Daily Living (B-ADL), combined with instruments for cognitive assessment such as the Mini-Mental State Examination (MMSE), applied to the informants, are recommended as the norm for application in Brazil.^7^ Several studies have suggested that the combination of a cognitive test with functional scales can improve the detection of dementia.^7,8^

Some studies published in the international literature have used the Direct Assessment of Functional Status (DAFS) by Loewenstein and Bates (1992) to assess the coherence between the informer and the functional capacity of the patient.^2-4^ The Pfeffer FAQ (1982) has also been used in another study as a predictive assessment based on self-report and on the informer’s report, regarding the functional deficit of patients with moderate cognitive impairment for the diagnostic follow-up of probable Alzheimer disease.^9^

In view of the above considerations, the objective of the present study was to compare the information provided by the caregiver, to the performance of the patient assessed by an examiner using the Pfeffer FAQ (1982).

## Method

### Subjects

Seventy-two patients and their caregivers participated in the study. The patients were attended by the Occupational Therapy Service of the Behavioral Neurology Outpatient Clinic during the period spanning from 1999 to 2001.

The inclusion criteria were: to have a confirmed diagnosis of dementia according to the DSM-IV, regardless of etiology or stage, to have attended three or more return visits, and to have a caregiver belonging to the family nucleus (spouse, daughter, son etc.).

Non-compliance with treatment was an important exclusion criterion since some patients and caregivers came to the Service for the initial evaluation and then abandoned treatment after one or two return visits. Another exclusion criterion was refusal to participate in the study. On the basis of these criteria, 47 subjects and their caregivers were excluded from the study.

Thus, a total of 25 patients followed up by the Occupational Therapy Service from 1999 to 2001 satisfied the inclusion criteria.

### Procedure

The study was approved by the Research Ethics Committee of the University Hospital, Faculty of Medicine of Ribeirão Preto, USP.

Two subject groups were studied: the CD GROUP, consisting of 25 patients with cognitive disorders, and the CG GROUP, consisting of 25 caregivers. The clinical and sociodemographic characteristics of the two groups are presented in Tables 1 and 2, respectively.

**Table 1:** Clinical and sociodemographic characteristics of patients.

Nº	Sex	Age	Schooling (years)	M.S.	Origin	Disease duration (years)	Diagnosis
1	M	59	4	D	Ribeirão Preto	7	Frontotemporal dementia
2	M	67	3	M	Ribeirão Preto	7	Dementia + Alcoholism
3	F	80	0	D	Ribeirão Preto	8	Alzheimer's disease
4	F	82	2	D	Brodosqui	6	Vascular dementia
5	F	75	11	M	Ribeirão Preto	9	Lewy body dementia
6	F	80	11	W	Batatais	8	Vascular dementia
7	M	45	5	M	Ribeirão Preto	9	Dementia syndrome+ Alcoholism
8	M	80	4	M	Ribeirão Preto	7	Dementia + Alcoholism
9	M	49	0	M	Guariba	14	Alzheimer's disease
10	M	46	5	S	Ribeirão Preto	3	Dementia + Hypoglycemia
11	F	73	1	S	Ribeirão Preto	5	Alzheimer's disease
12	F	66	8	M	Ribeirão Preto	5	Alzheimer's disease
13	M	66	3	M	Batatais	5	Alzheimer's disease
14	M	60	4	M	Ituverava	8	Vascular dementia
15	M	76	4	M	Brodosqui Ribeirão	13	Alzheimer's disease
16	F	86	0	W	Preto	5	Alzheimer's disease
17	M	58	3	M	Pradópolis Ribeirão	5	Dementia + Severe hypoxia
18	F	84	4	W	Preto	6	Alzheimer's disease
19	F	60	4	M	S. Carlos	5	Corticobasal dementia
20	F	86	1	M	Ribeirão Preto	4	Dementia + Severe hypoxia
21	F	86	0	W	Ribeirão Preto	4	Alzheimer's disease
22	F	71	3	W	Pontal	5	Vascular dementia
23	M	77	4	M	P. Ferreira	4	Vascular Dementia
24	M	29	2	S	Jaboticabal	4	Dementia + Alcoholism
25	F	75	12	W	Jardinópolis	4	Lewy Body Dementia

M.S., marital status; D, divorced; M, married; W, widowed; S, single; F, female; M, male.

**Table 2:** Sociodemographic characteristics of the major *caregivers of patients.

Nº	Caregiver's kinship	Caregiver's age	Caregiver's schooling (years)	Caregive R'S sex	Time AS caregiver (years)	Care time (hs/day)
1	Ex-Wife	55	11	F	7	12
2	Wife	67	2	F	7	12
3	Daughter	38	11	F	8	4
4	Daughter	55	8	F	6	4
5	Husband	75	15	M	9	12
6	Adopted daughter	48	8	F	8	12
7	Wife	46	4	F	9	12
8	Wife	72	4	F	7	12
9	Wife	43	4	F	14	12
10	Wife	52	1	F	3	12
11	Daughter	39	8	F	5	8
12	Husband	65	4	M	5	12
13	Wife	64	4	F	5	12
14	Wife	54	3	F	8	12
15	Wife	67	4	F	13	12
16	Nora	52	4	F	5	4
17	Wife	55	4	F	5	12
18	Daughter	55	17	F	6	4
19	Husband	64	4	M	5	4
20	Husband	89	1	M	4	12
21	Daughter	42	4	F	4	8
22	Son	43	3	M	5	4
23	Wife	74	4	F	4	12
24	Mother	75	1	F	4	8
25	Daughter-in-law	46	13	F	4	8

* Major or primary caregivers who accompanied the patients to visits and spent more time with the patient; F, female; M, male.

While an examiner interviewed the caregiver, another simultaneously evaluated the performance of the patient in the laboratory using the FAQ.

### Instrument applied to the caregiver

An examiner applied the FAQ^5^ to the caregivers, assigning scores from 0 to 3, allowing a maximum score of 30. The questionnaire was applied individually in an appropriate room protected in terms of secrecy and privacy. The examiner read out the questions and waited for the response of the caregiver. The caregiver was asked to provide information on the performance of his/her demented relative in tasks involving functions in the cognition area and instrumental activities of daily life. The examiner assigned the following scores to the information provided by the caregiver: zero (when the patient was able to perform the proposed task without difficulty), one (when the patient was able to perform the task but with difficulty), two (when the patient needed help to perform the task) or three (when the patient was unable to perform the proposed task). The sum of the scores obtained for each item gave the final score, which indicated the degree of limitation of the patient.

### Instrument applied to the patient

A laboratory was set up on the 3^rd^ floor of the University Hospital, Faculty of Medicine of Ribeirão Preto, USP, permitting the assessment of patient performance in simulated situations. The laboratory consisted of two rooms prepared as described below:

1. A simulated shopping situation was created in one of the rooms, with various objects left on view. The patient received a note pad and a pencil in order to organize his purchases, photocopies of blank checks, and money in bills and coins for payment.
2. In the room corresponding to the kitchen, the patient was asked to make coffee and to prepare a salad with a coffee “kit” (pot used to boil water, a coffee filter and jug, coffee powder, and sugar) and a salad “kit” (salad bowl, knife, fork, seasonings, a lettuce and tomato).

Another examiner asked the patient to perform the following FAQ activities: handling checks and money, organizing the chores for the day by verbalizing them or making written notes, buying objects in a simulated shopping situation, playing dominoes, cards or checkers, preparing simple food such as a green salad, watching a television program and commenting on what they saw, commenting on TV news, events regarding family and neighborhood, remembering to take their medications and return visits to the University Hospital using their hospital appointment card as an aid, and walking on the 3^rd^ floor, where they were supposed to locate the visiting room and the daily activities laboratory where the tests of the present study were performed, without getting lost along the way. Regarding this last item, care was taken to include patients who had attended more than three return visits, in order to ensure familiarity with the testing environment. The examiner assigned the following scores to the performance of the patient: **zero** (when the patient was able to perform the proposed task without difficulty), **one** (when the patient was able to perform the task but with difficulty), **two** (when the patient needed help to perform the task) or **three** (when the patient was unable to perform the proposed task).

The evaluations of the caregivers and of the patients were filed separately and kept secret until the end of the investigation. Data were analyzed statistically by the weighted Kappa coefficient and the non-parametric Wilcoxon test.

## Results

The clinical and sociodemographic characteristics of the patients who participated in the study were similarly distributed between males and females. Most subjects had four years of schooling, were married, were from Ribeirão Preto, and older than 71 years. Their diagnoses of dementia were as follows: Alzheimer disease, vascular dementia, frontotemporal dementia, dementia secondary to alcoholism, Lewy body degenerative dementia, corticobasal dementia, and dementia due to severe hypoxia. The course of the diseases ranged from 3 to 14 years (Table 1).

The main caregiver was the spouse, in most cases the wife, aged between 51 to 70 years with up to 8 years of schooling. Experience as principal caregiver ranged from 3 to 14 years and the time devoted to care was about 4 to 12 hours per day (Table 2).

Cognition and functionality were assessed during the course of treatment. Table 3 presents the cognitive and functionality profile during different stages, i.e., during treatment and the present investigation.

**Table 3:** Evaluation of cognition and functionality and dementia staging on first visit, and results of the evaluations performed over the study period.

Initial evaluation											Study period		
Medical scale					O.T.			Medical scale			Occupational therapy		
					1st caregiver			Date			Date	FAQ	FAQ
Id	MMSE	CDR	GDS		FAF	LAW		classification			O.T. evaluation	examiner	caregiver
1	13	2	5		10	23		15/08/01	Mo		14/08/01	23	29
2	16	3	6		26	63		07/06/01	S		15/08/01	25	30
3	11	2	5		21	69		01/08/01	Mo		22/08/01	20	22
4	5	3	5		25	54		21/11/01	S		23/01/02	21	27
5	27	1	3		6	46		05/04/02	Mi		04/09/01	4	15
6	3	3	6		26	46		14/03/01	S		11/09/01	30	29
7	11	3	7		27	63		20/02/02	S		15/08/01	23	30
8	24	0.5	2		11	39		21/03/01	Mi		22/08/01	3	21
9	10	3	6		20	54		05/12/01	S		05/12/01	20	30
10	13	1	2		21	57		30/08/00	Mi		05/09/01	25	30
11	5	3	6		11	32		01/08/01	S		18/09/01	16	20
12	9	2	5		15	42		17/10/01	Mo		05/09/01	10	21
13	3	3	7		30	84		15/08/01	S		15/08/01	30	30
14	21	2	5		19	42		05/12/00	Mo		12/09/01	2	21
15	20	0.5	2		8	38		15/08/01	Mi		26/09/01	7	26
16	14	2	6		16	54		05/12/00	Mo		15/08/01	23	22
17	14	1	5		24	55		15/08/01	Mo		10/10/01	22	30
18	20	2	5		15	45		18/10/00	Mo		22/08/01	5	23
19	12	1	4		10	29		08/08/01	Mi		01/08/01	11	22
20	12	2	5		16	55		12/04/02	S		07/01/01	25	30
21	23	1	2		0	5		20/02/02	Mi		10/10/01	5	1
22	9	2	5		15	45		03/10/01	Mo		19/09/01	12	25
23	14	3	6		28	55		23/01/02	S		12/09/01	24	25
24	7	2	5		19	53		19/09/01	Mo		19/01/01	14	25
25	26	3	5		27	67		17/10/01	S		19/03/01	9	30
26	21	3	7		26	69		26/03/02	S		08/08/02	7	28

Id, number of identification of the subjects in order studied; MMSE, Mini-Mental State Examination; CDR, Clinical Dementia Rating Scale; GDS, Global Deterioration Scale; FAQ, Pfeffer Functional Activities Questionnaire; LAW, Instrumental Activities of Daily Living - IADL by Lawton & Brody; Mo, moderate dementia; S, severe dementia; Mi, mild dementia; O.T., occupational therapy; FAQ examiner, Functional Activities Questionnaire applied to the patient which is judged and scored by the examiner; Caregiver FAF, Functional Activities Questionnaire applied to the caregiver.

Agreement between caregiver’s Pfeffer and examiner’s Pfeffer was studied (Table 4).

**Table 4:** Agreement between caregiver information, and investigator observations on functional performance of the patient in the laboratory of Instrumental Activities of Daily Life.

	Caregiver X Patient Agreement				Interpretation
	Weighted	SD			
Questions	K	(Weighted K)	P		
Q1 - Able to fill out a check, pay bills and handle money.	0.17	0.09	0.35		Weak and nonsignificant agreement
Q2 - Able to organize himself by making notes.	0.20	0.15	0.50		Weak and nonsignificant agreement
Q3 - Able to buy clothes and food.	0.48	0.15	0.78		Weak to reasonable and significant agreement
Q4 - Able to play cards, checkers and dominoes.	0.51	0.15	0.81		Weak to reasonable and significant agreement
Q5 - Able to make coffee and not to forget to turn off the stove.	0.34	0.15	0.64		Weak and nonsignificant agreement
Q6 - Able to prepare food.	0.21	0.10	0.31		Weak and nonsignificant agreement
Q7 - Able to keep up with community or neighborhood events.	0.24	0.11	0.35		Weak and nonsignificant agreement
Q8 - Able to pay attention to and to understand a television or ra-dio program and to read and understand a newspaper or magazine.	0.29	0.16	0.61		Weak and nonsignificant agreement
Q9 - Able to remember engagements, family events and his/ her medications.	-0.00	0.14	0.28		No agreement
Q10 - Able to take a walk outside the neighborhood without getting lost.	0.16	0.08	0.32		Weak and nonsignificant agreement

Data measured on the basis of the weighted Kappa coefficient.

Statistical analysis showed that out of ten items on the Pfeffer FAQ^5^ evaluated, the following seven showed weak and non-significant agreement: (1) handling money, (2) self-organization by making notes, (5) making coffee, (6) preparing food, (7) keeping up with community or neighborhood events, (9) reading the newspaper, and (10) taking a walk in the neighborhood without getting lost. Also, a weak and significant agreement was observed for items (3), shopping, and (4), playing cards and dominoes. No agreement was observed for item (9), which tested the ability of the patient to remember engagements, family events and medications.

Further analysis was conducted in order to compare the mean performance of the patient observed by the examiner, to the information provided by the caregiver, using the non-parametric Wilcoxon test for paired data (Table 5)

**Table 5:** Comparison between mean patient performance observed by the investigator and by the caregiver.

Questions	Caregiver				Patient			P	Interpretation
	Mean	SD Median			Mean	SD Median			
Q1 - Able to fill out a check, pay	2.73	0.83	3.00		1.73	1.31	2.00	0.002	Significant
bills and handle money.									Caregiver>Patient difference
Q2 - Able to organize him/herself	2.69	0.84	3.00		1.96	1.04	2.00	0.006	Significant
by making notes.									Caregiver>Patient difference
Q3 - Able to buy clothes and food.	2.62	0.80	3.00		2.08	0.93	2.00	0.002	Significant
									Caregiver>Patient difference
Q4 - Able to play cards, checkers	2.19	1.30	3.00		1.31	1.26	1.00	0.001	Significant
and dominoes.									Caregiver>Patient difference
Q5 - Able to make coffee and not to	2.23	1.18	3.00		1.31	1.12	1.50	0.002	Significant
forget to turn off the stove.									Caregiver>Patient difference
Q6 - Able to prepare food.	2.54	0.76	3.00		1.23	1.14	1.00	<0.001	Significant
									Caregiver>Patient difference
Q7 - Able to keep up with	2.62	0.90	3.00		1.58	1.14	2.00	0.001	Significant
community or neighborhood events.									Caregiver>Patient difference
Q8 - Able to pay attention to and	2.04	1.34	3.00		1.62	1.10	2.00	0.16	No significant
to understand a television or radio									Caregiver=Patient difference
program and to read and under-									
stand a newspaper or magazine.									
Q9 - Able to remember engage-	2.54	0.90	3.00		1.85	1.05	2.00	0.02	Significant
ments, family events and his/her									Caregiver>Patient difference
medications.									
Q10 - Able to take a walk outside the	2.65	0.85	3.00		1.35	1.23	1.50	0.001	Significant
neighborhood without getting lost.									Caregiver>Patient difference
Total	24.81	6.43	26.50		16.00	8.90	18.00	<0.001	Significant
									Caregiver>Patient difference

All items of the questionnaire, except item 8 (Table 5), presented significant differences between Pfeffer caregiver and Pfeffer examiner scores, where the caregiver tended to be more pessimistic when evaluating the patient, emphasizing the functional disability of the latter. In other words, from the viewpoint of the caregiver, the patient was more dependent.

## Discussion

The study showed weak and non-significant agreement for 7 items on the Pfeffer FAQ (1982) (1, 2, 5, 6, 7, 8 and 10), yet reasonable and significant agreement in items 3 and 4, and disagreement in item 9, i.e., there was disagreement regarding most of the items in the questionnaire.

In later analyses comparing mean patient performance observed by the examiner to the information provided by the caregiver, significant differences were observed in all items except item 8. The responses of the caregivers revealed that they were more pessimistic and tended to evaluate the performance of the patient in a more negative manner. The caregiver, when evaluating the patient, stressed the patient’s functional disability.

Similar studies^2-4^ have also detected a difference between the information provided by the caregiver and that drawn from direct patient evaluation in daily life activities. The reasons for this difference may be due to the following:

1) not all, but most of the caregivers tend to “do for the patient” instead of “doing with the patient”, thus masking the parameters of the response;
2) The examiner involved in the direct evaluation of functional performance was a health professional more attuned to determining the potential/limitations of the patient.

However, the present results differ from those reported in other studies.^2-4^ American and European caregivers tend to overestimate the ability of patients to perform daily life activities, whereas the caregivers studied in the present work underestimated the functional capacity of patients.

The differences detected may possibly be related to factors such as socioeconomic and cultural level which may strongly influence the assessment of the caregiver. Some studies have pointed out the compromise caused by dementia in the daily life of both patient and caregiver: the load and the stress due to direct patient care,^2-4^ as well as intra-family conflicts, financial difficulties, loss of purchasing power after retirement along with increased expenses involved in patient care, and lack of a social and health support network. All of these factors may contribute to the impairment of the physical and mental health of the caregiver.^10^ However, this relationship warrants further investigation.

One study concluded that depression interferes with the evaluation of the caregiver while care load does not,^2^ whereas others have stated the opposite for caregiver depression and load respectively.^4^

Some important limitations of the present study should be emphasized:

1. In the present approach both caregiver and examiner (a health professional) answered the FAQ. We suggest that a third evaluator trained for direct patient observation during the performance of the tasks be included in future studies.
2. The time taken for application of the FAQ tasks was one hour on average, a length of time considered unviable in daily clinical practice.
3. We suggest the inclusion in future studies of an instrument for screening caregivers regarding depression, quality of life and load, so that the results may be compared with those of previous studies.^2-4^
4. The present study was conducted on a specific group that frequents a behavioral neurology outpatient clinic, representing selected demand and thus prevents the generalization of the findings to the general sphere of Brazilian caregivers of patients with dementia.

Despite its limitations, the present study highlights the need to set up a program of continued guidance with information for the caregiver and relatives about dementia, as well as the need for future studies in order to better understand the attitude of the caregiver toward dementia. We can conclude that the information provided by the caregivers is unreliable in as far as the caregiver underestimates functional capacity of the patient and considers the patient to be more dependent than they really are.

## QUESTIONÁRIO DE ATIVIDADES FUNCIONAIS

1. Ele(a) é capaz de preencher cheques, pagar contas, manejar o próprio dinheiro?
  0 = Normal (ou: Nunca o fez, mas poderia fazê-lo agora)
  1 = Faz com dificuldade (ou: Nunca o fez e agora teria dificuldades)
  2 = Necessita ajuda
  3 = Não é capaz
2. Ele(a) é capaz de organizar suas coisa, fazer anotações?
  0 = Normal (ou: Nunca o fez, mas poderia fazê-lo agora)
  1 = Faz com dificuldade (ou: Nunca o fez e agora teria dificuldades)
  2 = Necessita ajuda
  3 = Não é capaz
3. Ele(a) é capaz de comprar roupas, comida, coisa para casa, sozinho?
  0 = Normal (ou: Nunca o fez, mas poderia fazê-lo agora)
  1 = Faz com dificuldade (ou: Nunca o fez e agora teria dificuldades)
  2 = Necessita ajuda
  3 = Não é capaz
4. Ele(a) é capaz de jogar baralho, dama, dominó, etc?
  0 = Normal (ou: Nunca o fez, mas poderia fazê-lo agora)
  1 = Faz com dificuldade (ou: Nunca o fez e agora teria dificuldades)
  2 = Necessita ajuda
  3 = Não é capaz
5. Ele(a) é capaz de esquentar água para um café sem se esquecer de apagar o fogo?
  0 = Normal (ou: Nunca o fez, mas poderia fazê-lo agora)
  1 = Faz com dificuldade (ou: Nunca o fez e agora teria dificuldades)
  2 = Necessita ajuda
  3 = Não é capaz
6. Ele(a) é capaz de preparar uma comida?
  0 = Normal (ou: Nunca o fez, mas poderia fazê-lo agora)
  1 = Faz com dificuldade (ou: Nunca o fez e agora teria dificuldades)
  2 = Necessita ajuda
  3 = Não é capaz
7. Ele(a) é capaz de manter-se em dia com os acontecimentos atuais da comunidade ou vizinhança?
  0 = Normal (ou: Nunca o fez, mas poderia fazê-lo agora)
  1 = Faz com dificuldade (ou: Nunca o fez e agora teria dificuldades)
  2 = Necessita ajuda
  3 = Não é capaz
8. Ele(a) é capaz de prestar atenção e entender um programa de rádio ou televisão, um jornal ou uma revista?
  0 = Normal (ou: Nunca o fez, mas poderia fazê-lo agora)
  1 = Faz com dificuldade (ou: Nunca o fez e agora teria dificuldades)
  2 = Necessita ajuda
  3 = Não é capaz
9. Ele(a) é capaz de lembrar-se de compromissos, acontecimentos familiares, seus medicamentos?
  0 = Normal (ou: Nunca o fez, mas poderia fazê-lo agora)
  1 = Faz com dificuldade (ou: Nunca o fez e agora teria dificuldades)
  2 = Necessita ajuda
  3 = Não é capaz
10. Ele(a) é capaz de passear fora da vizinhança sem se perder?
  0 = Normal (ou: Nunca o fez, mas poderia fazê-lo agora)
  1 = Faz com dificuldade (ou: Nunca o fez e agora teria dificuldades)
  2 = Necessita ajuda
  3 = Não é capaz
    Anote a pontuação ............... (máx. 30)

## References

[1] Brasil, Ministério da Saúde Programas e Projetos: saúde do idoso.

[2] Arguelles S, Loewenstein DA, Eisdorfr C, Arguelles T (2001). Caregiver's judgments of the functional abilities of the
Alzheimer's disease patient: impact of caregivers' depression and perceived
burden. J. Geriatr Psychiatry Neurol.

[3] Loewenstein DA, BATES CB (1992). The direct assessment of functional status (DAFS). Manual for
Administration and Scoring.

[4] Zanetti O, Geroldi C, Frisoni GB, Bianchetti A, Trabuchi M (1999). Contrasting results between caregiver's report and direct
assessment of activities of daily living in patients affected by mild and
very mild dementia: the contribution of the caregiver's personal
characteristics. J Am Geriatr Soc.

[5] Pfeffer RI, Kurosaki TT, Harrah CH, Chance JM, Filos S (1982). Measurement of functional activities in older adults in the
community. J. Gerontology, Orange.

[6] Herrera E Jr, Caramelli P, Silveira AS, Nitrini R (2002). Epidemiologic survey of dementia in a community-dwelling
Brazilian population. Alzheimer Dis Assoc Disord.

[7] Nitrini R, Caramelli P, Bottino CMC, Damasceno BP, Brucki SD, Anghinah R (2005). Diagnóstico de Doença de Alzheimer.
Avaliação Cognitiva. Recomendações do
Departamento Científico de Neurologia Cognitiva e do Envelhecimento
da Academia Brasileira de Neurologia. Arq Neuropsiquiatr.

[8] Bustamante SE, Bottino CM, Lopes MA (2003). Combined instruments on the evaluation of dementia in the
elderly: preliminary results. Arq Neuropsiquiatr.

[9] Tabert MH, Albert SM, Borukhova-Milov L (2002). Functional deficits in patients with mild cognitive impairment -
Prediction of AD. Neurology.

[10] Santos SMA (2003). Idoso, família e cultura: um estudo sobre a
construção do papel do cuidador.

